# Associations of loneliness and social isolation with cardiovascular and metabolic health: a systematic review and meta-analysis protocol

**DOI:** 10.1186/s13643-020-01369-8

**Published:** 2020-05-04

**Authors:** Adriano Winterton, Linn Rødevand, Lars T. Westlye, Nils Eiel Steen, Ole A. Andreassen, Daniel S. Quintana

**Affiliations:** 1 NORMENT, KG Jebsen Centre for Psychosis Research, Division of Mental Health and Addiction, University of Oslo and Oslo University Hospital, Building 49, Ullevål, Kirkeveien 166, PO Box 4956, N-0424 Oslo, Nydalen Norway; 2 Department of Psychology, University of Oslo, Oslo, Norway

**Keywords:** Loneliness, Social isolation, Meta-analysis, Metabolic syndrome, Mental illness, Cardiovascular risk

## Abstract

**Background:**

A growing number of studies suggest that social isolation and loneliness are associated with premature mortality and are more prevalent among people with mental illness than in the general population, outlining many potential paths to disease still to be elucidated. The purpose of this meta-analysis is to examine the relationship between loneliness, social isolation, and established cardiovascular/metabolic risk factors and disorders, especially in severe mental illness, and to account for potential heterogeneity in the literature.

**Methods/design:**

Studies that report measures of loneliness and/or social isolation along with cardiovascular/metabolic risk factors will be identified. PubMed, EMBASE (through Ovid SP), Scopus, and PsycINFO (through Ovid SP) will be searched, along with citation lists of retrieved articles and the Cochrane Database of Systematic Reviews. Grey literature will be searched using Google Scholar. Data will be extracted from eligible studies for a random effects meta-analysis. For each study, a summary effect size, heterogeneity, risk of bias, publication bias, and the effect of categorical and continuous moderator variables will be determined.

**Discussion:**

This proposed systematic review and meta-analysis will identify and synthesise evidence to determine if there is an association between loneliness, social isolation, and cardiovascular/metabolic risk factors, with a special focus on severe mental illnesses. The results will help determine links and promising avenues of further research.

**Systematic review registration:**

PROSPERO CRD42018111911

## Background

A growing number of studies suggest that social isolation and loneliness are associated with general morbidity and premature mortality [1, 2] and are more prevalent among people with severe mental illness than in the general population [3], outlining many potential paths to disease [4–7]. Loneliness has been described as the distressing subjective experience of lacking relationships or missing a certain level of quality in them [8]. Social isolation, on the other hand, concerns the objective characteristics of one’s relationships and refers to shortcomings in the size of their social network [9]. Although the relationship between loneliness and social isolation is complex [9], both have been associated with mental health and cardiometabolic disease and mortality [10], particularly among the elderly. Furthermore, although people with severe mental illness (SMI) experience high levels of both loneliness [11, 12] and cardiometabolic disease [13], little is known about the relationship between loneliness and social isolation and cardiometabolic health in patients with SMI, and to which degree such associations may be related to the reduced life expectancy in patients with SMI [13, 14]. Thus, a better understanding of the relationships between loneliness and social isolation and cardiometabolic health is needed, both in the general population and in patients with SMI. To this end, we will conduct a meta-analysis of studies on loneliness, social isolation, and their associations with cardiovascular and metabolic risk, building on the methodology of a previous systematic review and meta-analysis of the role of social isolation and loneliness as risk factors for coronary heart disease and stroke [15]. Compared to the previous publication [15], the present meta-analysis has a broader scope by looking at more general cardiometabolic conditions, including risk factors and some diseases of the cardiovascular system, as well as a special attention to SMI.

## Methods/design

### Aims

The aim of the present meta-analysis is to examine the relationship between loneliness, social isolation, and established cardiovascular/metabolic risk factors and disorders, and to elucidate the possible mechanisms in which these two measures of quality and quantity of interpersonal relationships can affect metabolic and cardiovascular health, with special regard to these relations in SMI. We focus on these associations in the general population and in patients with SMI. This protocol has been registered in PROSPERO (registration number CRD42018111911) and is outlined in agreement with the PRISMA-P guidelines [16] (Additional file 1).

### Search strategy

**Fig. 1:**
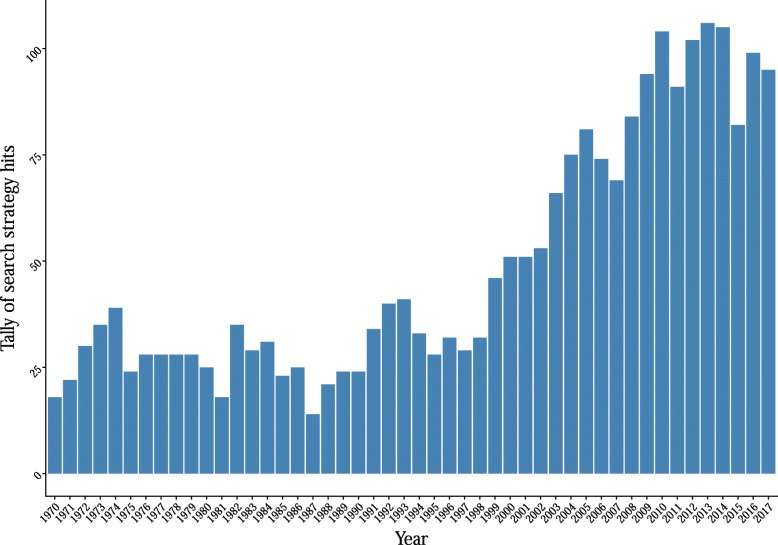
Number of hits per year of a focused search strategy in PubMed. Scripts to generate the illustration are available at this link: https://osf.io/p2sb3/ [17]

We will conduct a systematic literature search to identify studies that evaluate the relationship between measures of loneliness and social isolation and established cardiovascular and metabolic risk factors. After consultation with an academic librarian, the first searches will be performed in PubMed, EMBASE (through Ovid SP), Scopus, PsycINFO (through Ovid SP), the Cochrane Database of Systematic Reviews, and Google Scholar with a search based on the following terms: loneliness/or social isolation/or social deprivation/or social alienation/or psychosocial deprivation/. This search strategy was independently proofread by a second academic librarian at the University of Oslo. It should be noted that this is a partial snippet of the entire, more comprehensive, search strategy (Additional file 2). Bibliographies of review articles found from this search will be consulted for relevant citations and subsequently reference lists within studies and citing articles of selected studies will be examined for remaining studies that could be relevant. There will be no limitations based on publication period or language (Fig. 1).

### Inclusion and exclusion criteria

Prospective cohorts, retrospective cohorts, and case-control studies will be included in the study if they include measurements of loneliness and/or social isolation and some measure of cardiovascular and metabolic risk and disease, as described below.

#### Loneliness

Measures used in included studies should be consistent with the definition of loneliness as a subjective negative feeling associated with someone’s subjective perception that their relationships with others are quantitatively and/or qualitatively deficient (e.g. the de Jong Gierveld Loneliness Scale, the UCLA Loneliness Scale [8]). We anticipate that many studies will not use tools that exclusively measure loneliness, so other tools will also be considered: (a) tools where loneliness is not identified as the concept being measured alone, but where questions or subscores might fit the definition of loneliness above (e.g. the Multidimensional Scale of Perceived Social Support [18], the Duke-UNC Functional Social Support Questionnaire [19]); (b) single-item measurement tools, though these may present unique challenges, as participants’ understanding of the concept may be different. Some researchers have also suggested that, given the stigma associated with loneliness, a direct, single question, such as: “do you feel lonely”, is not appropriate for capturing people’s feelings of loneliness [15]. To avoid discarding potentially useful information, studies with this type of measurement will not be excluded from this review, their findings will be analysed separately to explore how they might differ from those associated with other approaches to measurement, and the type of test will be included as a factor when estimating bias and included as a moderator.

#### Social isolation

Measures used in included studies should be consistent with social isolation as an objective measure of the lack of relationships, ties, or contacts with other people (e.g. The Lubben Social Network Scale and its shortened version [20, 21]). Few tools and studies explicitly measure social isolation; therefore, the search strategy includes numerous terms relating to interpersonal interaction (see supplement). Studies that only include questions focusing on the presence or absence of a specific relationship (e.g. marital status) will not be included, as the definition of social isolation used in this review is broader.

#### Loneliness and social isolation

Some tools combine items relating to loneliness and some items relating to social isolation (e.g. the Older Americans Resources and Services Social Resource Scale [22]). Studies that used such tools will be included in the review, and a subgroup analysis will be performed to look at how findings reported using these tools compare with tools focusing on either of the two elements.

#### Reliability and validity of the measures

Studies will not be excluded based on the reliability and validity of the tools used to measure loneliness or social isolation. Subgroup analyses will be carried out to explore the relationship between the choice of measurement tools and the effects reported, and bias will be estimated for each.

#### Type of measure

The types of measures used are expected to vary and are likely to include dichotomous (e.g. lonely vs. not lonely) and continuous (e.g. score on loneliness scale) measures. The type of measure used will be taken into account when extracting and synthesising the data but will not constitute a criterion for exclusion.

#### Cardiovascular and metabolic risk and disease

Studies will be included if they report cardiovascular or metabolic risk factors and diseases/outcomes. We define a risk factor as a measurable element or characteristic that is causally associated with an increased rate of a disease and that is an independent and significant predictor of the risk of presenting a disease [23]. In the case of cardiovascular and metabolic disorders, these have been the focus of innumerable studies in the past decades, given that they are the greatest cause of death globally [24] and their incidence has steeply increased worldwide [25], with the decrease in high-income countries counterbalanced by the increase in low- and middle-income ones. We plan on including both outcomes (stroke, diabetes, MetS, CHD) and known risk factors (hypertension, BMI and other indices of obesity, dyslipidaemia, inflammation markers [where applicable], smoking, and physical activity).

Titles and abstracts of studies retrieved using the search strategy and those from additional sources will be screened independently by two independent reviewers (AW and LR) to identify studies that potentially meet the inclusion criteria. The full text of these studies will be retrieved and independently assessed for eligibility by two review team members. Any disagreement between them over the eligibility of studies will be resolved through discussion with a third reviewer (DQ).

### Moderators

Some study and population factors will be considered in the analysis—age, gender ratio, type of obesity index used, mental health status, somatic health status, medications, socio-economic status, ethnicity and/or region, specific diagnostic criteria used for metabolic syndrome, loneliness, and social isolation models—as independent factors or linked factors. The mental health status will be used for subgroup analyses to give a more accurate picture in SMI. As mentioned above, the specific tool used to measure social isolation and/or loneliness will also be used as a moderator, as well as risk of bias and year of publication.

### Quality assessment (risk of bias)

Two review authors will independently assess bias risk for each of the included studies using the RTI item bank [26], which evaluates selection bias, confounding, performance bias, detection bias, attrition bias, and selective outcome reporting. Any discrepancies in assessment will be assessed and resolved by a third author.

Small study bias will be evaluated through the use of funnel plots and Egger’s test; publication bias will be assessed with the p-uniform* method using the “puniform” tool by van Aert [27] (https://rdrr.io/github/RobbievanAert/puniform/man/puniform.html).

### Data extraction and management

Two independent reviewers (AW and LR) will extract data from all eligible studies using the data extraction form (Additional file 3). This form includes general information on studies including authors, title, number of effect sizes, sample size, and effect sizes with regard to stroke, coronary heart disease, diabetes, hypertension, BMI, metabolic syndrome, smoking, and physical activity; information about the participants including gender, age, physical and mental health status, and ethnicity or location of the study; information on other moderator variables, including study type, type of scale used (for loneliness, social isolation, or both), diagnostic criteria used (for diabetes, metabolic syndrome, etc.), the model used for loneliness and social isolation and their potential interaction, and adjustment for potential confounders; and information concerning study quality including publication year and the risk of bias measures as defined by the RTI item bank.

### Statistical analysis

All statistical analyses will be performed using the *metafor* [28] and *puniform* [27] packages in R. Summary effect sizes will be obtained from effect sizes and sample size of each study, with correlations transformed to Fisher’s *z*. The modality of extraction will depend on the individual study. If effect sizes are reported in the publication, these will be transformed to Fisher’s *z*; if the effect sizes are not reported, but individual results are, then *r* will be obtained and converted to *z*. For studies published within the last 15 years that do not report the effect sizes numerically, authors will be contacted and asked to provide, if possible, that information. For studies published earlier than 15 years ago, or whose authors do not respond, and that report results in scatterplot graphs, a conversion to numerical values will be performed using a plot digitiser (https://automeris.io/WebPlotDigitizer/). The numerical values will then be used to calculate *r* and transformed into *z*. Studies for which it is not possible to perform any of the above will not be included into the meta-analysis. The Grading of Recommendations, Assessment, Development and Evaluations (GRADE) framework will be used to assess the strength of the body of evidence.

### Power analysis

We performed a power analysis following the equations of Valentine et al. [29], using a custom script (https://osf.io/k8jth/ [30]) to estimate the ranges of effect sizes that this meta-analysis would be suitable to detect, using average number per group (*n* = 764) and average number of effects (*n* = 6) calculated from a previous meta-analysis [31]. Given the expected effect size (0.2) at medium levels of heterogeneity, we would achieve at least 99% power. Even if these values were adjusted conservatively (effect size of 0.1 and high heterogeneity), statistical power remains above 99%.

### Summary effect size

Summary effect sizes will be computed assuming a random effects model, as the effect sizes of individual studies are expected to vary substantially due to differences in populations. In case this is not true, the random effects model collapses to a fixed effect model, so there is no loss of information. In addition, in the presence of heterogeneity, relative weights are more balanced than those assigned under fixed effects, as standard random effects methods add a common component of variance to each study weight to account for between-study variability in treatment effects. Consequently, this double source of variability (within and between study) will lead to wider variance, standard error, and CI for the summary effect.

### Sample heterogeneity

The *Q*-statistic is the weighted sum of the products of squared differences between study effect sizes and summary effect sizes. As such, it is a measure of the total observed dispersion of the estimated effect sizes. A significant *Q*-statistic is indicative of significantly different effect sizes between the studies included in the meta-analysis. In this meta-analysis, we follow the convention of an alpha level of .05 for the *Q*-statistic.

Calculated on *Q*, the *I*^2^ statistic expresses the proportion of the total dispersion that accounts for true dispersion, being the ratio between the excess of dispersion and total dispersion. It is not the estimate of an underlying amount of heterogeneity but only a descriptive statistic, rather it is a measure of inconsistency among the findings of the studies, and it is not affected by the number of studies included in the meta-analysis. The *I*^2^ statistic is useful for determining whether the amount of real effect size variance between studies is relatively higher than chance variability. The *Q*-statistic, the significance of the *Q*-statistic, and *I*^2^ will be computed and reported.

### Cluster-robust analysis

When studies provide more than one effect size, these are statistically dependent from each other, forming groups of internally correlated effect size estimates. Conventional meta-analytical techniques rely on the assumption that the effect size estimates from different studies are independent when this is not true, adjustments need to be performed. If the covariance of the effect size is known, it can be included in models to adjust for this [32].

Without access to original datasets, however, the covariance between effect size estimates of included studies is rarely available, as covariances are seldom reported; moreover, assuming a fixed covariance value can lead to errors in effect size estimation. Cluster-robust meta-analyses can account for statistically dependent clusters without assuming a fixed covariance value when covariances are not reported [32]. As such, cluster-robust meta-analysis will be applied to assess outcomes. A random mixed-effects meta-analysis assuming a diagonal v matrix will be used to construct cluster-robust models for each outcome.

### Moderator analysis

For each categorical moderators, a random effects model will be applied, calculating summary mean effects for each subgroup. The inter-study variation metric *T*^2^ will be obtained for each subgroup, and means will then be compared using a two-tailed *z*-test in order to determine the probability for the difference of observed means given equality of true means, assuming normal distribution. A *p* value below .05 will be seen as indicative of moderating properties of the variable in question. A comparison of the effect sizes calculable for these subgroups will be performed in such cases.

For the continuous moderator variables (age, risk of bias, and year of publication), meta-regression will be performed to estimate an unstandardized regression coefficient along with the coefficient’s significance level.

## Discussion

Research on the association between loneliness and social isolation and health has increased over the last decade, with growing evidence regarding associations to overall mortality [6, 15]. The goal of this meta-analysis is to give a clearer picture of the mechanisms that connect loneliness/social isolation and cardiometabolic health, with a particular focus on this connection in SMI.

The mechanisms by which loneliness and social isolation impact health outcomes are unclear for a number of reasons, including that [4] social isolation is associated with general morbidity and mortality rather than with the aetiology of any specific disease, [5] the term “social isolation” can be applied to numerous kinds of social relations (e.g. spousal relationship, membership in clubs, contacts with friends), and the effects of social relationships on long-term morbidity and mortality take years to unfold and many measurements of loneliness do not take this into account, specifically in cross-sectional studies [4]. It is also unclear whether the association between social relationships and cardiometabolic health varies according to age, sex, socioeconomic status, mental health, and somatic health. This meta-analysis aims to fill these gaps with a more nuanced look at the specifics of these associations.

Loneliness and social isolation are also of great interest with regard to the cardiovascular mortality tied to mental illness [4]. Loneliness appears to be heavily determined by genetics, personality, and cognition of the individual [33, 34]. It has been shown to be deleterious for mental health as well as physical health, and a bidirectional causal relation between loneliness and social cognition has also been proposed [35, 36]. Impaired social cognition, a common trait of many types of mental illness, is thought to play a causal role in the experience of loneliness [34, 37].

## Conclusion

A more nuanced overview of the specific cardiovascular mortality and morbidity risks tied to loneliness and social isolation are needed to understand and clarify the underlying biological mechanisms. Several possible pathways to disease have been proposed [4, 38, 39], involving sleep quality, effect on lifestyle habits, and homeostatic changes. Importantly, given the high prevalence of loneliness among people with mental health problems and the evidence for its harmful effects in other populations [40], it is important to analyse these associations in SMI. A promising connection between loneliness, social isolation, and homeostatic regulation is oxytocin, due to its involvement in metabolic regulation, social functioning, and feeding behaviours [38, 39, 41–45], though some of the findings are from animal models. This meta-analysis hopes to shed light on this and become a solid foundation for further studies on these underlying pathways.

## Supplementary information

**Additional file 1:.** PRISMA-P (Preferred Reporting Items for Systematic Review and Meta-Analysis Protocols) 2015 checklist. Recommended items to address in a systematic review protocol.

**Additional file 2:.** Complete search strategy.

**Additional file 3:.** Data extraction form. Record of pertinent study characteristics for each included study.

## Data Availability

Not applicable
