# Supplementary material for: Associations of loneliness and social isolation with cardiovascular and metabolic health: a systematic review and meta-analysis protocol
Source: Syst Rev. 2020 May 4;9:102. doi: 10.1186/s13643-020-01369-8 (PMC7199368; doi:10.1186/s13643-020-01369-8)
Supplement: Supplementary file 2 — Additional file 2:. Complete search strategy. [file 13643_2020_1369_MOESM2_ESM.pdf]

Protocol

Search strategy

We will search the following electronic databases to identify relevant studies:

- Medline (Ovid 1946 to current)
- EMBASE (Ovid 1974 to current)
- PsycINFO (Ovid 1806 to current)
- Scopus (Elsevier)
- Cochrane Database of Systematic Reviews
- Google Scholar

We will not limit our searches by language or year of publication.

Primary studies

In the search strategy for primary studies, we will use search filters to identify case-control, cross-sectional and cohort studies.

Systematic reviews

Relevant systematic reviews will be identified, and we will ensure that any relevant primary studies included in these reviews are retrieved and considered for inclusion. The systematic reviews search will be limited to Ovid Medline, Embase, and Cochrane Database of Systematic Reviews.

Search for primary studies:

Ovid Medline

1. loneliness/ or social isolation/ or social deprivation/ or social alienation/ or psychosocial deprivation/
2. (loneliness or lonely or social isolation or socially isolated or social alienation or psychosocial deprivation or social deprivation).ti,ab,kw
3. exp social support/ or social participation/ or social environment/ or social capital/ or family relations/ or friends/ or interpersonal relations/ or social distance/
4. (social support or social network or social networks or social participation or social relationship* or social capital or social interaction* or friends or friendship* or family relation* or interpersonal relation* or social distance or (social and disconnect*) or living alone).ti
5. (risk factor or risk factors or associat* or impact* or affect* or influenc* or related).ti
6. 1 or 2 or ((3 or 4) and 5)

7. (Animal Experimentation/ or exp Animals/ or exp Models, Animal/) not Humans/
8. ((veterinary or animal or animals or rabbit or rabbits or rodent or rodents or rat or rats or mouse or mice or pig or pigs or dog or dogs or cat or cats) not (patient or patients or human or humans)).ti.
9. 6 not (7 or 8)
10. Epidemiologic studies/
11. Exp case control studies/
12. Exp cohort studies/
13. Case control.tw.
14. (cohort adj (study or studies)).tw.
15. Cohort analy$.tw.
16. (Follow up adj (study or studies)).tw.
17. (observational adj (study or studies)).tw.
18. Longitudinal.tw.
19. Retrospective.tw.
20. Cross sectional.tw.
21. Cross-sectional studies/
22. Or/10-21
23. 9 and 22

In Ovid Medline, we use a search filter for identifying observational studies, as described in: http://resourcecenter.ovid.com/site/resources/expert_search/healthexp.html#OvidFilters

Embase

1. loneliness/ or social isolation/ or social alienation/
2. (loneliness or lonely or social isolation or socially isolated or social alienation or psychosocial deprivation or social deprivation).ti,ab,kw
3. exp social support/ or social participation/ or social environment/ or social capital/ or family relation/ or friend/ or social interaction/
4. (social support or social network or social networks or social participation or social relationship* or social capital or social interaction* or friends or friendship* or family relation* or interpersonal relation* or social distance or (social and disconnect*) or living alone).ti
5. (risk factor or risk factors or associat* or impact* or affect* or influenc* or related).ti
6. 1 or 2 or ((3 or 4) and 5)
7. (exp animal/ or exp animal model/ or nonhuman/) not exp human/
8. ((veterinary or animal or animals or rabbit or rabbits or rodent or rodents or rat or rats or mouse or mice or pig or pigs or dog or dogs or cat or cats) not (patient or patients or human or humans)).ti.
9. 6 not (7 or 8)

10. Clinical study/
11. Case control study/
12. Family study/
13. Longitudinal study/
14. Retrospective study/
15. Prospective study/
16. Randomized controlled trials/
17. 15 not 16
18. Cohort analysis/
19. (Cohort adj (study or studies)).mp.
20. (Case control adj (study or studies)).tw.
21. (follow up adj (study or studies)).tw.
22. (observational adj (study or studies)).tw.
23. (epidemiologic$ adj (study or studies)).tw.
24. (cross sectional adj (study or studies)).tw
25. or/10-14, 17-24
26. 9 and 25

In Embase, we use a search filter for identifying observational studies, as described in: http://resourcecenter.ovid.com/site/resources/expert_search/healthexp.html#OvidFilters

PsycINFO

1. loneliness/ or social isolation/ or social deprivation/
2. (loneliness or lonely or social isolation or socially isolated or social alienation or psychosocial deprivation or social deprivation).tw
3. ((social support or social network or social networks or social participation or social relationship* or social capital or social interaction* or friends or friendship* or family relation* or interpersonal relation* or social distance or (social and disconnect*) or living alone) and (risk factor or risk factors or associat* or impact* or affect* or influenc* or related)).ti
4. (1 or 2 or 3)
5. ((veterinary or animal or animals or rabbit or rabbits or rodent or rodents or rat or rats or mouse or mice or pig or pigs or dog or dogs or cat or cats) not (patient or patients or human or humans)).ti.
6. 4 not 5
7. (cohort study or cohort studies or cohort analysis or cohort analyses or longitudinal or prospective or retrospective or case control or follow up study or follow up studies or observational study or observational studies or cross sectional).tw
8. 6 and 7

Scopus

Advanced:

(TITLE-ABS-KEY (loneliness OR lonely OR "social isolation" OR "socially isolated" OR "social alienation" OR "psychosocial deprivation" OR "social deprivation") OR TITLE (("social support" OR "social network" OR "social networks" OR "social participation" OR "social relationship" OR "social relationships" OR friends OR friendship* OR "family relation" OR "family relations" OR "family relationship" OR "family relationships" OR "interpersonal relation" OR "interpersonal relations" OR "interpersonal relationship" OR "interpersonal relationships" OR "social distance" OR (social AND disconnect*) OR "living alone") AND ("risk factor" OR "risk factors" OR associat* OR impact* OR affect* OR influenc* OR related))) AND TITLE-ABS-KEY ("cohort study" OR "cohort studies" OR "cohort analysis" OR "cohort analyses" OR longitudinal OR prospective OR retrospective OR "case control" OR "follow up study" OR "follow up studies" OR "observational study" OR "observational studies" OR "cross sectional") AND NOT (TITLE (veterinary OR animal OR animals OR rodent OR rodents OR rat OR rats OR mouse OR mice OR dog OR dogs OR cat OR cats) AND NOT TITLE (patient OR patients OR human OR humans))

Google Scholar

allintitle: (loneliness|lonely|"social isolation"|"socially isolated" |"social alienation" |"psychosocial deprivation" |"social deprivation") AND (cohort|"case control" |longitudinal |retrospective |"cross sectional")

Search for systematic reviews:

Ovid Medline

1. loneliness/ or social isolation/ or social deprivation/ or social alienation/ or psychosocial deprivation/
2. (loneliness or lonely or social isolation or socially isolated or social alienation or psychosocial deprivation or social deprivation).ti,ab,kw
3. exp social support/ or social participation/ or social environment/ or social capital/ or family relations/ or friends/ or interpersonal relations/ or social distance/
4. (social support or social network or social networks or social participation or social relationship* or social capital or social interaction* or friends or friendship* or family relation* or interpersonal relation* or social distance or (social and disconnect*) or living alone).ti
5. (risk factor or risk factors or associat* or impact* or affect* or influenc* or related).ti
6. 1 or 2 or ((3 or 4) and 5)

7. (Animal Experimentation/ or exp Animals/ or exp Models, Animal/) not Humans/
8. ((veterinary or animal or animals or rabbit or rabbits or rodent or rodents or rat or rats or mouse or mice or pig or pigs or dog or dogs or cat or cats) not (patient or patients or human or humans)).ti.
9. 6 not (7 or 8)
10. Limit 9 to "systematic review"
11. (systematic review or meta-analys* or metaanalys*).ti
12. 9 and 11
13. 10 or 12

Embase

1. loneliness/ or social isolation/ or social alienation/
2. (loneliness or lonely or social isolation or socially isolated or social alienation or psychosocial deprivation or social deprivation).ti,ab,kw
3. exp social support/ or social participation/ or social environment/ or social capital/ or family relation/ or friend/ or social interaction/
4. (social support or social network or social networks or social participation or social relationship* or social capital or social interaction* or friends or friendship* or family relation* or interpersonal relation* or social distance or (social and disconnect*) or living alone).ti
5. (risk factor or risk factors or associat* or impact* or affect* or influenc* or related).ti
6. 1 or 2 or ((3 or 4) and 5)
7. (exp animal/ or exp animal model/ or nonhuman/) not exp human/
8. ((veterinary or animal or animals or rabbit or rabbits or rodent or rodents or rat or rats or mouse or mice or pig or pigs or dog or dogs or cat or cats) not (patient or patients or human or humans)).ti.
9. 6 not (7 or 8)
10. meta analysis/ or "systematic review"/
11. (systematic review or meta-analys* or metaanalys*).ti
12. 10 or 11
13. 9 and 12

Cochrane Database of Systematic Reviews

#1 MeSH descriptor: [Social Isolation] explode all trees
#2 MeSH descriptor: [Loneliness] explode all trees
#3 MeSH descriptor: [Social Alienation] explode all trees
#4 MeSH descriptor: [Psychosocial Deprivation] explode all trees
#5 (loneliness OR lonely OR "social isolation" OR "socially isolated" OR "social alienation" OR "psychosocial deprivation" OR "social deprivation"): ti,ab,kw

#6 #1 OR #2 OR #3 OR #4 OR #5
#7 MeSH descriptor: [Social Support] explode all trees
#8 MeSH descriptor: [Social Participation] explode all trees
#9 MeSH descriptor: [Social Environment] explode all trees
#10 MeSH descriptor: [Family Relations] this term only
#11MeSH descriptor: [Friends] explode all trees
#12 MeSH descriptor: [Interpersonal Relations] this term only
#13 MeSH descriptor: [Social Distance] explode all trees
#14 ((social AND (support OR network* OR participation OR relationship* OR capital OR interaction*)) OR friends OR friendship* OR ((family OR interpersonal) AND relation*) OR "social distance" OR (social AND disconnect*) OR "living alone"): ti
#15 #7 OR #8 OR #9 OR #10 OR #11 OR #12 OR #13 OR #14
#16 ((risk AND factor*) OR associat* OR impact* OR affect* OR influenc* OR related): ti
#17 #15 AND #16
#18 #6 OR #17
