# Supplementary material for: Associations of loneliness and social isolation with cardiovascular and metabolic health: a systematic review and meta-analysis protocol
Source: Syst Rev. 2020 May 4;9:102. doi: 10.1186/s13643-020-01369-8 (PMC7199368; doi:10.1186/s13643-020-01369-8)
Supplement: Supplementary file 3 — Additional file 3:. Data extraction form. Record of pertinent study characteristics for each included study. [file 13643_2020_1369_MOESM3_ESM.pdf]

Categories	Values
A. Identification of study	
1. Study ID	
2. Authors	
3. Title	
4. Year of Publication	
B. Primary Values	
1. Number of Correlations	
2. Stroke	
3. Coronary Heart Disease	
4. Diabetes	
5. Hypertension	
6. BMI (Obesity indicator)	
7. Dyslipidaemia	
8. Metabolic Syndrome	
9. Inflammation Markers (if applicable)	
10. Smoking	
11. Physical activity	
C. Population	
1. Gender (%)	
2. Age range	
3. Sample size	
4. Mental Health Status	
5. Somatic Health Status	
6. Ethnicity\Region	
D. Moderators	
1. Study type	
2. Scale used (loneliness/Social isolation assessment)	
3. Criteria used	
4. Loneliness x social isolation models	
5. Adjustment for potential confounders/covariates	
E. Bias	
1. Questionnaire	
